# Rope Jumping Strength Monitoring on Smart Devices via Passive Acoustic Sensing

**DOI:** 10.3390/s22249739

**Published:** 2022-12-12

**Authors:** Xiaowen Hou, Chao Liu

**Affiliations:** Sanya Oceanographic Institution, College of Computer Science and Technology, Ocean University of China, Qingdao 266100, China

**Keywords:** acoustic sensing, domain adversarial adaptation, generative adversarial network, passive rope jump monitoring, short-term energy

## Abstract

Rope jumping, as a fitness exercise recommended by many sports medicine practitioners, can improve cardiorespiratory capacity and physical coordination. Existing rope jump monitoring systems have limitations in terms of convenience, comfort, and exercise intensity evaluation. This paper presents a rope jump monitoring system using passive acoustic sensing. Our system exploits the off-the-shelf smartphone and headphones to capture the user’s rope-jumping sound and breathing sound after exercise. Given the captured acoustic data, the system uses a short-time energy-based approach and the high correlation between rope jumping cycles to detect the rope-jumping sound frames, then applies a dual-threshold endpoint detection algorithm to calculate the number of rope jumps. Finally, our system performs regression predictions of exercise intensity based on features extracted from the jumping speed and the mel spectrograms of the user’s breathing sound. The significant advantage of the system lies in the solution of the problem of poorly characterized mel spectrograms. We employ an attentive mechanism-based GAN to generate optimized breathing sound mel spectrograms and apply domain adversarial adaptive in the network to improve the migration capability of the system. Through extensive experiments, our system achieves (on average) 0.32 and 2.3% error rates for the rope jumping count and exercise intensity evaluation, respectively.

## 1. Introduction

Nowadays, with the increasing pressures of life and work, maintaining physical health and improving physical fitness have become hot topics of widespread concern. Some medical experts say that rope jumping can improve cardiorespiratory functions [1], enhance physical fitness, and soothe emotions [2]. Studies have confirmed that rope jumping could prevent diseases, such as diabetes, arthritis, osteoporosis, hypertension, depression, and many others. This is the reason why doctors recommend rope jumping as a form of daily exercise. However, most rope jumpers do not know their own exercise intensities and lack the reference of the training load in the actual exercise [3,4], so they cannot scientifically monitor and evaluate their own rope-jumping exercises. Improper exercise intensity may damage one’s health and even result in unnecessary injuries or death in some specific cases [5]. In addition, over-training can lead to excessive fatigue and loss of motivation for exercise. Thus, the awareness of exercise intensity is of great importance to achieve optimal training outcomes. To facilitate exercise health monitoring, researchers have come up with various solutions. Fitness monitoring based on acoustic signals is preferred over vision-based solutions [6,7,8] with lighting requirements and privacy as risks. Wearable device-based fitness monitoring systems [9,10,11,12,13] allow for accurate classification and counting of fitness movements. However, these systems require the user to wear additional equipment when exercising, which places additional physical burdens on the user, and these systems do not monitor the intensity of the user’s exercise. Therefore, researchers are beginning to investigate device-free fitness monitoring systems.

Smart speaker-based acoustic sensing technology [14] turns smart speakers into active sonars for fitness monitoring, but it can only monitor in an indoor environment with a smart speaker and the users cannot change their positions while using it. The Wi-Fi-based approaches [15,16] cannot be performed outdoors where there is no Wi-Fi signal or a weak Wi-Fi signal, and the Wi-Fi signal is susceptible to interference from the surrounding environment. Furthermore, all of the systems/products mentioned above mainly focus on recognizing the type of exercise being performed or counting the number of exercise repetitions and do not have the capability to perform exercise intensity monitoring.

Unlike the aforementioned work, we aim to perform jump rope counting and exercise intensity monitoring by leveraging the user’s rope-jumping sound and breathing sound captured by the headphone, which is readily available with almost all smartphones. Our proposed system does not require special rope-jumping equipment or rope jumping environment and it has strong anti-interference capabilities.

**Figure 1:**
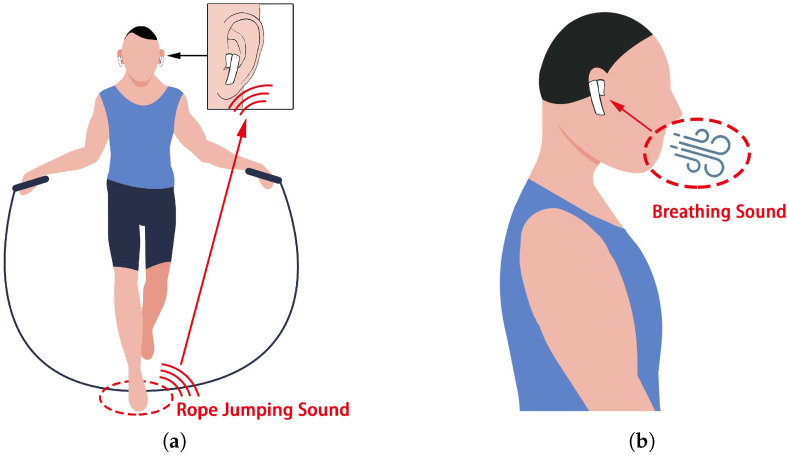
Application scenarios. (**a**) Capturing the rope jumping sound for rope jumping counting. (**b**) Capturing the breathing sound for evaluating exercise intensity.

In order to achieve this aim, we propose a system that uses headphones to capture acoustic signals for jump rope counting and exercise intensity monitoring. Our system adopts headphones to capture the sound of the user’s foot hitting the ground when rope jumping (the rope-jumping sound), as shown in Figure 1a, and then relies on the rope-jumping sound to count jump ropes, and captures the user’s breathing sound after rope jumping to evaluate the user’s exercise intensity, as shown in Figure 1b. Smartphone headphones are used extensively during exercises [17]. Although existing rope jump monitoring systems can rely on the Apple Watch, according to the market researcher Counterpoint Research, only about 1.42% of the world’s population is equipped with an Apple Watch, while close to 80% of people use headphones in their daily lives. It is, thus, possible for us to explore utilizing the smartphone’s headphones to monitor rope jumping. The key idea of this system is to use the high correlation between rope jumping cycles to count the rope jumping and to evaluate the intensity of the rope jumping exercise based on the difference in the user’s breathing after the exercise. However, there are multiple challenges to implementing the system. First, due to the fact that the rope-jumping sound is intermittent, the collected acoustic signal usually contains many other sounds or silent fragments. The system should be able to accurately identify the sound segment corresponding to the rope-jumping sound. Second, the presence of ambient noise causes part of the breathing sound to be captured unclearly, resulting in a poorly characterized mel spectrum generated from the breathing sound. Third, because of the diversity of users and usage scenarios, the system needs to have some migration capability to adapt to different users and environments.

To address the above challenges, we first pre-process the captured signal to remove most of the ambient noise. We then apply a short-time energy-based method to detect the rope-jumping sounds and leverage the high correlation between rope jumping cycles to further improve the accuracy of the rope-jumping sound detection. We implement jump rope counting based on a dual-threshold endpoint detection algorithm and calculate the rope jumping speed. In the evaluation phase of exercise intensity, we design an attentive mechanism-based GAN to generate optimized breathing mel spectrograms and then take the optimized breathing mel spectrograms and rope jumping speed as inputs to extract features for regression prediction of exercise intensity. To enable the system to adapt to different users and environments, domain-adaptive adaptation is incorporated into the network, which allows the system to extract a rich set of domain-independent features. Our contributions are summarized as follows:

- We propose a rope jump monitoring system; this is the first work that successfully introduces attentive mechanism-based GAN to acoustic sensing. Such an optimization strategy improves the utilization of the collected data so that some audio data that are not recorded clearly can also be fully utilized and effectively improve the prediction accuracy of the system. It also avoids the over-reliance on rope-jumping equipment and environments (as conducted in previous works), and adds exercise intensity evaluation to the basic counting function; hence, it significantly broadens the scope of real-world applications.
- We designed a robust method for the detection of the rope-jumping sound by exploiting the properties of the high short-time energy of the rope-jumping sound and the high correlation between the rope jumping cycles.
- We designed a neural network that incorporates an attentive mechanism-based GAN and domain-adaptive adaptation to improve the prediction accuracy of the rope jumping exercise intensity and migration capability of the system.
- We conducted extensive experiments with eight volunteers in different environments to evaluate our system. The results show that the system can perform rope jump monitoring with, on average, 0.32 and 2.3% error rates for rope jumping count and exercise intensity evaluation, respectively.

## 2. Related Work

Based on recent works, we summarize related work in the following four aspects.

### 2.1. Research in Fitness Movement Monitoring

Many researchers monitor fitness movements by leveraging various devices. FEMO [11] applies the Doppler shift to monitor exercise by attaching passive RFID tags on the dumbbells. RecoFit [12] can automatically track repetitive exercises via an arm-worn inertial sensor. MiLift [13] uses commercial off-the-shelf smartwatches to accurately track both cardio and weightlifting workouts. However, these methods require the user to wear additional equipment, which places an additional burden on the user and only allows for the classification and counting of fitness movements, not for fine-grained assessment of exercise intensity.

### 2.2. Research in Exercise Intensity Monitoring

There are some works dedicated to exercise intensity monitoring. Wu et al. [8] propose to monitor sports training intensity based on a camera-based heart rate detection algorithm and a fatigue expression feature extractor. However, such camera-based methods require high lighting conditions and have privacy issues. Pernek et al. [18] leverage the smartphone’s acceleration stream to detect individual resistance training repetitions and provide feedback about the quality of exercises based on the duration of an individual repetition. However, using this method requires the user to wear the phone on their arm or attach it to gym equipment, which places an additional burden on the user and allows the system to be used only in fixed situations. Calorie Map [16] utilizes wireless signals to infer calories burned while exercising and monitors activity intensity. However, this method requires the user to work out within the Wi-Fi signal coverage and the Wi-Fi signal can easily be interfered with.

### 2.3. Research in Adversarial Learning

Our system utilizes attentive mechanism-based GAN for spectrogram optimization. Recently, various GAN models have been used for compositing and translating images. When applying GANs to image restoration and enhancement, most existing works use paired training data as well, SRGAN [19] leverage GAN for image super-resolution (SR) and propose a perceptual loss function, which consists of an adversarial loss and a content loss to recover photo-realistic textures. Shuai Yang et al. [20] present shape-matching GAN to transfer text styles, with a scale-controllable module designed to allow for adjusting the stylistic degree of the glyph with a continuous parameter as user input and generating diversified artistic text in real-time. However, all of these approaches require paired training data, and it is impractical to simultaneously capture pairs of image data of the same visual scene. Several unsupervised GANs are proposed to learn inter-domain mappings using adversarial learning and are adopted for many other tasks. Jun-Yan Zhu et al. [21] adopted a two-way GAN to translate between two different domains by using a cycle-consistent loss with unpaired data. A handful of the latest works followed their methodology and applied unpaired training with cycle consistency to several low-level vision tasks, e.g., mobile photo enhancement. Our system is different as it adopts a lightweight one-path GAN structure, which is stable and easy to train.

### 2.4. Research in Rope Jump Monitoring

Our system is a smartphone-based rope jump monitoring system. There have been active studies on rope jump monitoring. Smart Rope [22] designed a smart jump rope that has a sensor inside the handle to count the jump ropes. However, this method requires the user to use a fixed jumping tool and can only record the number of jumps without assessing the intensity of the exercise. YaoYao [23] uses the motion sensor in the Apple Watch to count jump ropes, measure the speed of the jump, and give the user a heart rate record. However, this method can only be used with the Apple Watch and the system only gives a record of the user’s heart rate; there is often a time delay of several tens of seconds [24] between the change in heart rate and the actual intensity of the exercise, making it difficult for the users to assess their exercise intensities based on their heart rate. TianTian [25] is a vision-based method that requires the user to use a camera to record the entire movement for jump rope counting. However, this method is heavily influenced by lighting and does not have the ability to monitor exercise intensity.

Unlike the above works, we aim to perform rope jumping intensity monitoring by leveraging the user’s rope-jumping sound captured by the headphone, which almost all smartphones are equipped with. Our system is not limited by environmental conditions and is also easy to use without the need to wear any additional sensors.

## 3. System Overview

The basic idea of our system is to use headphones to collect the rope-jumping sound and breathing sounds for the rope jumping intensity assessment.

**Figure 2:**
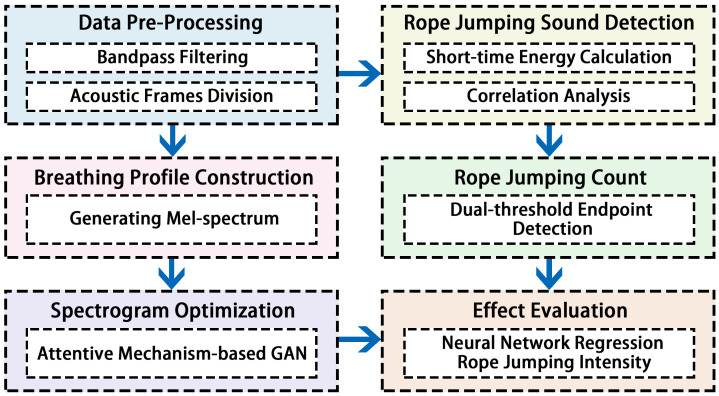
System architecture.

Figure 2 shows the architecture of our system. The system consists of six parts, which are data pre-processing, rope-jumping sound detection, rope jumping count, breathing profile construction, spectrogram optimization, and effect evaluation. We use wireless headsets to collect the sound while rope jumping and then pre-process the sampled acoustic signal. Specifically, we adopt a band-pass filter on the sampled signal and frame the signal. Once the high-frequency noise is removed, the system performs rope-jumping sound detection. We divide the signal into three segments. For each segment of the signal, the short-time energy of each signal frame is calculated to detect the rope-jumping sound and we further improve the rope jumping detection accuracy by exploiting the high correlation between rope jumping cycles.

Regarding the rope jumping count, we applied the dual-threshold endpoint detection algorithm to calculate the number of rope jumping counts and further calculate the average rope jumping speed. We collect the user’s breathing sound after rope jumping, then generate mel spectrograms in the breathing profile construction and optimize the breathing sound mel spectrograms in spectrogram optimization with an attentive mechanism-based GAN. Finally, users usually care about exercise effects, to ensure that physical fitness is improved. So the effect evaluation aims to assess the rope jumping strength. In detail, the optimized mel spectrogram and the calculated average rope jumping speed are sent to the network to extract features and regress the exercise intensity, which includes an adaptive domain discriminator to adapt to different users and environments.

### 3.1. Data Pre-Processing

Data pre-processing aims to clean the recorded rope-jumping sound by removing the noise components. In our work, we used headphones to capture the acoustic signal and extract the rope-jumping sound to detect the user’s rope-jumping movement. When rope jumping outdoors or at the gym, the rope-jumping sound is easily interfered with by environmental noise (e.g., the sound of talking, walking, etc.). In addition, the equipment used to collect the rope-jumping sound also produces a subtle equipment noise [26]. We adopted a band-pass filter to remove the sound components with high or low frequencies that are irrelevant to the rope jumping events. Specifically, the recorded sound signal was segmented into multiple frames with equivalent lengths *L* = 800. Then we applied the band-pass filter with lower and upper cutoff frequencies (at 500 Hz and 8000 Hz) to each frame for noise reduction. The lower cutoff frequency of 500 Hz could filter the thermal noise at a lower frequency band, while the upper cutoff frequency of 8000 Hz ensures that most rope jumping-related sound components are included.

### 3.2. Rope-Jumping Sound Detection

After data pre-processing, our system aims to detect and separate the rope-jumping sound from the acoustic signal for accurate jump rope counting. We find that when the user skips with headphones on, the energy of the frames containing the rope-jumping sound is higher than that of the other frames, so we calculate the short-time energy of each frame and detect the rope-jumping sound rope based on the short-time energy.

The short-time energy is often used as a basis for distinguishing between voiced and unvoiced speech segments. Assuming that the signal is sampled into *N* samples, which is X(n), 1 ⩽ *n* ⩽ *N*, and then divided into fn frames, the signal of the *i*-th frame is represented as:

(1) $$y_{i}\left(l\right)=X\left(\left(i-1\right)inc+l\right),1\;⩽\;l\;⩽\;L,\;1\;⩽\;i\;⩽\;fn,$$

where *L* is the frame length and inc is the frame shift. We define the energy of the *i*-th frame as:

(2) $$E\left(i\right)=\frac{1}{L}\sum_{l=1}^{L}y_{i}^{2}\left(l\right),1\;⩽\;i\;⩽\;fn.$$

**Figure 3:**
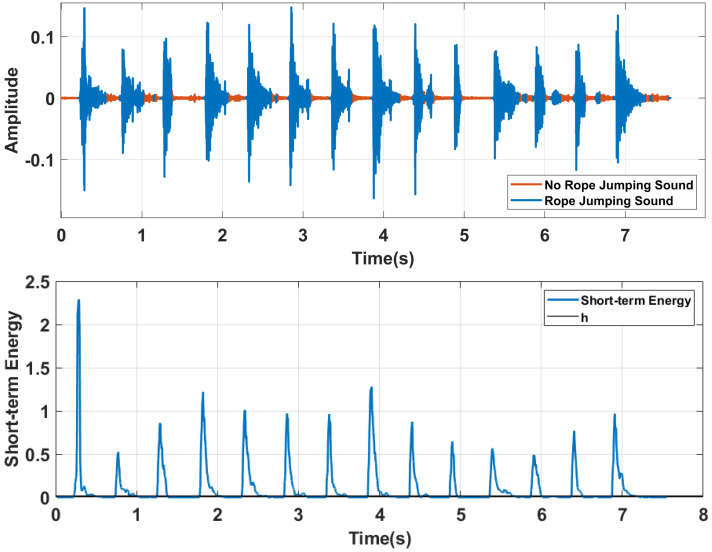
An example of the rope−jumping sound detection.

Figure 3 shows the time domain diagram of the rope-jumping sound and the corresponding short-time energy.

In order to extract the desired sound, we set a dynamic threshold based on the short-time energy [27,28]. Specifically, the histogram of the short-time energy sequence is first calculated, and then the histogram is smoothed. Finally, we detect the local maxima of the histogram. Let $H_{1}$ and $H_{2}$ be the locations of the first and second largest local maxima, respectively. The threshold *h* can be calculated as follows:

(3) $$h=\frac{\omega H_{1}+H_{2}}{\omega +1},$$

ω is the weight parameter, the larger the ω is, the closer the threshold will be to $H_{1}$. In this system, through the experimental analysis, we chose ω = 6 to make the best results. Then we made the parts of the signal energy below the threshold zero and obtain the processed signal as $X{\left(n\right)}^{\prime }$. Figure 3 shows the result of the threshold calculation; the black line in the figure is the threshold.

The parts of the signal energy below the threshold are represented in the orange color in Figure 3, we can see that the rope-jumping sound could be correctly identified.

**Figure 4:**
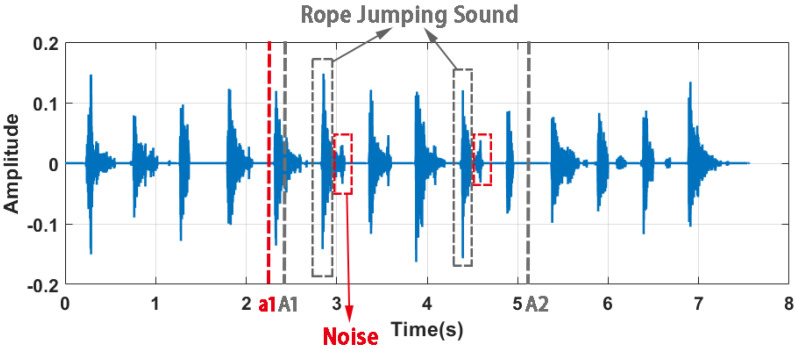
An example of splitting signals.

The detected segments are not directly representative of the rope-jumping sound as there is also noise, as shown in Figure 4. To accurately count the number of rope jumps, it is necessary to separate the reliable rope-jumping sounds. Figure 3 shows the signal collected by the headset. We can observe that rope-jumping movements can be considered cyclical over a short period of time. Based on this observation, we exploited the high correlation between the sound of each skip to detect the rope-jumping sound.

Specifically, considering that the user’s rope jumping speed may change slightly, we first split the collected signal into three segments, the points of division are $A_{1}$ and $A_{2}$, respectively. To protect the desired sound from being split, if $X{\left(A_{1}\right)}^{\prime }$≠ 0, then it is traversed from $A_{1}$ until the $a_{1}$ is obtained and $X{\left(a_{1}\right)}^{\prime }$ = 0, $a_{1}$ is used as the new segmentation point for the signal; the same goes for $A_{2}$. An example of splitting signals is shown in Figure 4. It can be seen that $A_{1}$ splits from the rope-jumping sound signal, which does not meet our expectations, and $a_{1}$ is the new split point.

We used the high correlation between two rope-jumping sounds to obtain the jumping period and then generated a square wave sequence based on the period as a simulated rope jumping sequence. We calculated the correlation between the simulated rope jumping sequence and the real rope jumping sequence for different parameters; the simulated jump rope sequence with the highest correlation is more representative of the user’s jump rope rhythm [29].

Specifically, we used the time *t* corresponding to the first non-zero frame in each signal segment as the start time of the square wave sequence. Let $T_{min}$ and $T_{max}$ be the minimum and maximum values of the number of frames occupied by the user in the rope jumping cycle, respectively. According to the experiment, $T_{min}$ = 6 frames and $T_{max}$ = 25 frames. We define a function f(j) on the period of the rope jumping to measure the correlation between two rope-jumping sounds at the period T=j:

(4) $$f\left(j\right)=\sum_{i=1}^{N-LT_{max}}{\left(X\left(i\right)-X\left(i+j\right)\right)}^{2},LT_{min}\;⩽\;j\;⩽\;LT_{max}.$$

A smaller value of f(j) indicates a higher correlation. We then find the value of *j* that minimizes the value of f(j) as the user’s rope jumping period *T*:

(5) $$T=\arg\min_{j}f(j),LT_{min}\;⩽\;j\;⩽\;LT_{max}.$$

**Figure 5:**
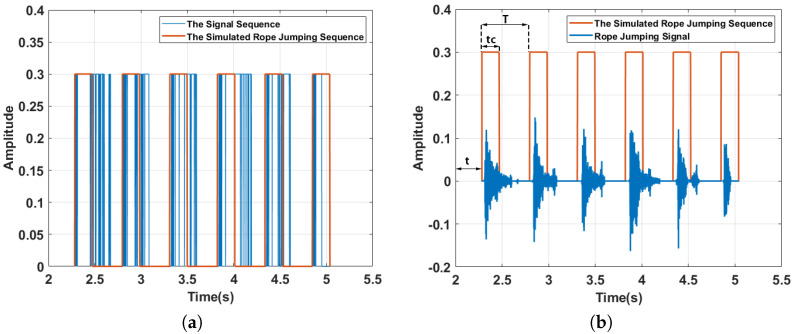
An example of generating the simulated rope jumping sequence. (**a**) Comparison of two generated sequences. (**b**) rope−jumping sound detection.

We generated the corresponding square wave sequence $S=S_{n},n=1,\ldots ,N$ based on the detected rope-jumping sound $X{\left(n\right)}^{\prime }$:

(6) $$S_{n}=\left\{\begin{array}{c} 0.3\;X{\left(n\right)}^{\prime }\neq 0\\ 0\;X{\left(n\right)}^{\prime }=0 \end{array}\right.,n=1,\ldots ,N,$$

and generated a simulated rope jumping sequence $S_{n}^{\prime }=S_{n}^{\prime }\left(tc\right)$ based on the derived *t* and *T*; tc is the number of frames in a period of the square wave that is greater than zero. Figure 5a shows the result of generating a sequence using the middle segment of the signal as an example, the blue line is the signal-based-generated sequence and the orange line is the simulated rope jumping sequence.

The correlation between the two square wave sequences $S_{n}$ and $S_{n}^{\prime }$ can be calculated as:

(7) $$\begin{array}{cc} C & =Corr\left\{S_{n},S_{n}^{\prime }\left(tc\right)\right\}\\ \; & =\frac{\sum_{n=1}^{N}\left(S_{n}-\bar{S_{n}}\right)\left(S_{n}^{\prime }\left(tc\right)-\bar{S_{n}^{\prime }\left(tc\right)}\right)}{\sqrt{\sum_{n=1}^{N}{\left(S_{n}-\bar{S_{n}}\right)}^{2}}\sqrt{\sum_{n=1}^{N}{\left(S_{n}^{\prime }\left(tc\right)-\bar{S_{n}^{\prime }\left(tc\right)}\right)}^{2}}}. \end{array}$$

We can calculate the value of tc that maximizes the value of *C*:

(8) $$T_{c}=\arg\max_{tc}C,0\;⩽\;tc\;⩽\;T.$$

Finally, based on the calculated *t*, *T*, Tc, we generate a square wave sequence $S_{n}^{\prime }$ that best represents the rhythm of the user’s rope jumping. Figure 5b shows the final generated simulated rope jumping sequence. We can see that the simulated rope jumping sequence basically corresponds to the rope-jumping sound.

We apply the above method to each segment of the signal, generating separately the square wave sequence corresponding to that segment, and finally stitching the three generated square wave sequences together.

The signal is then processed according to the spliced square wave sequence:

(9) $$X{\left(n\right)}^{\prime \prime }=\left\{\begin{array}{cc} X{\left(n\right)}^{\prime }\;S_{n}^{\prime } & =0.3\\ 0\;S_{n}^{\prime } & =0 \end{array}\right.,n=1,\ldots ,N,$$

If $S_{n}^{\prime }$ = 0.3, the corresponding original signal is considered to contain the rope-jumping sound, otherwise, the corresponding original signal is considered to be noise. $X{\left(n\right)}^{\prime \prime }$ is the separated rope-jumping sound.

### 3.3. Rope Jumping Count

In order to accurately count the number of rope jumps and calculate rope jumping speed, we segmented each rope-jumping sound. In other words, we needed to detect the start and end of each rope-jumping sound. We used a dual-threshold endpoint detection algorithm to segment each rope-jumping sound [30].

The algorithm uses a combination of short-time energy and short-time past zero rates, using both short-time energy and short-time past zero rate thresholds to determine the start and end points of the rope-jumping sound. Before starting the endpoint detection, we first set a smaller start threshold and a larger start threshold for the short-time energy and the short-time past zero rate, respectively. If the energy or over-zero rate of a frame X(i) exceeds the low threshold, it is marked as the starting point, and if the energy or over-zero rate of the signal in the next three frames exceeds the high threshold, then X(i) is determined as the starting point of the signal.

**Figure 6:**
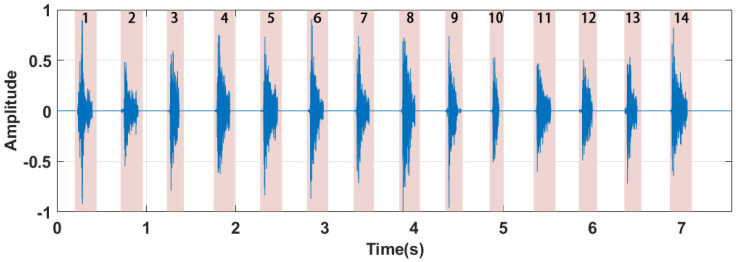
Separation result.

We then search for the corresponding end point of the signal in the same way. Then we continue to search for the start and end points of the next segment of the rope-jumping sound until the end of the signal. Figure 6 shows the result of the separation; we can see that all start and end points are correctly identified. The number of signal segments is the number of rope jumps $N_{jump}$. At the end of rope jumping, the user’s jump speed *V* is calculated as follows:

(10) $$V=\frac{N_{jump}}{T_{jump}},$$

where $T_{jump}$ is the time for rope jumping. If the user suddenly stops for more than two jumping cycles, it is assumed that the user has made a mistake or taken a break, at which point, the recording stops.

### 3.4. Breathing Profile Construction

**Figure 7:**
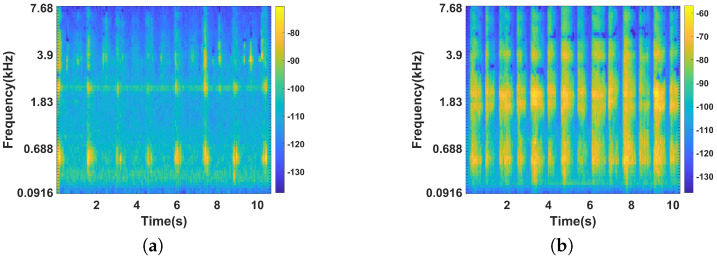
Comparison of breathing sound spectrograms at different exercise intensities. (**a**) Low exercise intensity. (**b**) High exercise intensity.

To monitor the rope jumping intensity, we used headphones to capture the user’s breathing sound within 30 s of the end of the exercise. Research has proven that breathing is an effective indicator of a person’s level of fatigue during exercise and is closely related to the intensity of the user’s exercise [31,32]. Figure 7 shows the spectrum of the user’s breath at different exercise intensities. We can observe that the breathing sound lies mostly in the low-frequency range and the breathing sound at a higher exercise intensity has higher energy than that at lower exercise intensity. We then applied a band-pass filter with upper and lower cutoff frequencies of 100 Hz and 3500 Hz, respectively, for noise reduction, ensuring that most of the noise was removed while retaining most of the breath-related sound. Research has shown that humans do not perceive frequencies linearly and are more sensitive to low-frequency signals than high-frequency signals. For example, we can detect the difference between 500 Hz and 1000 Hz easily, but it is difficult for us to detect the difference between 7500 Hz and 8000 Hz. Thus, we use the mel spectrogram to capture this characteristic. The mel spectrogram is a commonly used audio processing method and is now widely used in speech recognition, audio denoising, and other fields. Specifically, we first obtained the time-frequency spectrogram of the breathing sound signal using the short-time Fourier transform (STFT). STFT is essentially a windowed Fourier transform, which is defined as follows:

(11) $$STFT_{z}=\int_{-\infty }^{+\infty }z\left(u\right)g\left(u-t\right)e^{-j2\pi fu}du,$$

where *z* is the breathing sound signal, *f* is the frequency, *g*(*u*-*t*) is the window function, and *u* is the half window size of time *t*.

Because of the relatively large size of the spectrogram generated by the short-time Fourier transform, in order to obtain the sound feature of the proper size, it is generally transformed into a mel spectrogram by passing through a mel scale filter bank. To convert the ordinary frequency scale into the mel frequency scale *m*, we use the following equation:

(12) $$m=2595*\log_{10}\left(1+\frac{f}{700}\right).$$

After the transformation, we obtain the breathing sound mel spectrogram.

### 3.5. Spectrogram Optimization

**Figure 8:**
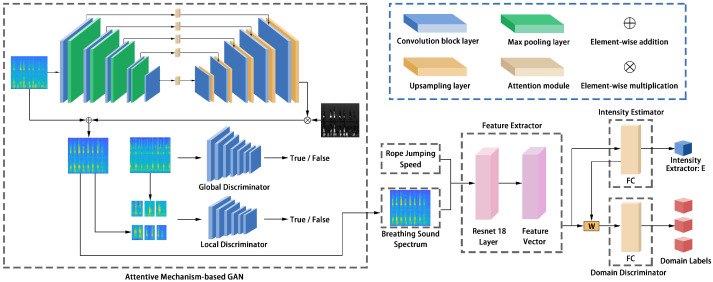
Network architecture.

The existence of noise in the environment and the presence of an inhomogeneous breathing sound result in the directly generated breathing mel spectrograms not being distinctly characterized. To further improve the prediction accuracy, we optimized the generated mel spectrograms using an attentive mechanism-based GAN. As shown in Figure 8, the GAN consists of two main parts: a generator *G* and a discriminator *D*. The generator consists of an attention-guided U-Net, and the discriminator part adopts a combination of global and local discriminators for improving the problem of obscure global and clear local features in the breathing mel spectrogram.

#### 3.5.1. U-Net Generator

U-Net is a U-shape network that connects the encoding layer to the decoding layer and helps information to flow correctly from the encoder to the decoder. U-Net has achieved huge success in image restoration and enhancement [33]. We, thus, adopt U-Net as our generator backbone. We further introduce the attention mechanism and add attention modules to each layer of the encoder and decoder connection. In order to highlight the features of the breathing mel spectrogram, we take the red channel Red of the input mel spectrogram, normalize it to [0,1], and then use *R* as our attention map. We then resize the attention map to fit each feature map and multiply it with all intermediate feature maps.

#### 3.5.2. Global-Local Discriminators

Directly generated breathing sound mel spectrograms often suffer from poorly characterized local areas, and the use of only one global discriminator is not capable of adaptively optimizing local areas. Therefore, we used a combination of global and local discriminators, both using PatchGAN for real/fake discrimination. PatchGAN can retain the detailed information of the spectrogram well, and it consists of convolutional layers with a final output matrix, for which the final true or false output is obtained by taking the mean value. For the global discriminator, we utilize the relativistic discriminator structure, which estimates the probability that real data are more realistic than fake data and also directs the generator to synthesize a fake spectrogram that is more realistic than real spectrograms. The function of a relativistic discriminator is:

(13) $$D_{R_{a}}\left(x_{real},x_{fake}\right)=\sigma \left(C\left(x_{real}\right)-E_{x_{fake}∼P_{fake}}\left[C\left(x_{fake}\right)\right]\right),$$

(14) $$D_{R_{a}}\left(x_{fake},x_{real}\right)=\sigma \left(C\left(x_{fake}\right)-E_{x_{real}∼P_{real}}\left[C\left(x_{real}\right)\right]\right),$$

where *C* denotes the network of the discriminator, $x_{real}$ and $x_{fake}$ are sampled from the real and fake distributions, and σ represents the sigmoid function. We used the least-square GAN (LSGAN) [34] loss to replace the sigmoid function in the relativistic discriminator. Therefore, the loss functions for the global discriminator *D* and the generator *G* are:

(15) $$\begin{array}{cc} L_{D}^{Global}= & E_{x_{real}∼P_{real}}\left[{\left(D_{R_{a}}\left(x_{real},x_{fake}\right)-1\right)}^{2}\right]+\\ & E_{x_{fake}∼P_{fake}}\left[{\left(D_{R_{a}}\left(x_{fake},x_{real}\right)\right)}^{2}\right], \end{array}$$

(16) $$\begin{array}{cc} L_{G}^{Global}= & E_{x_{fake}∼P_{fake}}\left[{\left(D_{R_{a}}\left(x_{fake},x_{real}\right)-1\right)}^{2}\right]+\\ & E_{x_{real}∼P_{real}}\left[{\left(D_{R_{a}}\left(x_{real},x_{fake}\right)\right)}^{2}\right]. \end{array}$$

The local discriminator learns to distinguish whether they are real (from real spectrograms) or fake (from optimized outputs) by taking randomly cropped local patches from both the output and real spectrograms and adopting the original LSGAN as the adversarial loss:

(17) $$\begin{array}{cc} L_{D}^{Lobal}= & E_{x_{real}∼P_{realpatches}}\left[{\left(D\left(x_{real}\right)-1\right)}^{2}\right]+\\ & E_{x_{fake}∼P_{fakepatches}}\left[{\left(D\left(x_{fake}\right)-0\right)}^{2}\right], \end{array}$$

(18) $$L_{G}^{Lobal}=E_{x_{real}∼P_{fakepatches}}\left[{\left(D\left(x_{fake}\right)-1\right)}^{2}\right].$$

Most of the GAN-based image enhancement networks require paired data, i.e., unclear breathing sound-transformed mel spectrograms and clear breathing sound-transformed mel spectrograms for the same exercise intensities, but the realistic spectrograms obtained often do not have the corresponding spectrogram data. In order to more effectively optimize the breathing sound mel spectrogram, the training and testing of the spectrogram optimization model (by taking unpaired spectrograms) require limiting the feature distance between the input spectrogram and the optimized output spectrogram. In order to more effectively optimize the breathing sound mel spectrogram, taking unpaired spectrograms for training and testing of the spectrogram optimization model, it is necessary to limit the feature distance between the input spectrogram and the optimized output spectrogram to preserve the spectrogram content features to itself, before and after the optimization. Thus, the loss can be defined as:

(19) $$L_{SFP}\left(I\right)=\frac{1}{W_{i,j}H_{i,j}}\sum_{x=1}^{W_{i,j}}\sum_{y=1}^{H_{i,j}}{\left(F_{i,j}\left(I\right)-F_{i,j}\left(G\left(I\right)\right)\right)}^{2},$$

where *I* denotes the input and G(I) denotes the output. $W_{i,j}$ and $H_{i,j}$ are the dimensions of the extracted feature maps. $F_{i,j}$ denotes the feature map extracted from the pre-trained VGG model. *i* represents its *i*-th max pooling, and *j* represents its *j*-th convolutional layer after the *i*-th max pooling layer.

Furthermore, a similar feature-preserving loss is used in the local patch of the local discriminator. We added an instance normalization layer [35] after each feature map before feeding into $L_{SFP}$ and $L_{SFP}^{Local}$ in order to stabilize training. Thus, the training loss of the GAN module can be expressed as:

(20) $$L_{GAN}=L_{SFP}^{Local}+L_{SFP}^{Global}+L_{G}^{Local}+L_{G}^{Global}.$$

### 3.6. Effect Evaluation

The effect evaluation aims to provide the user with a reliable estimate of the exercise intensity. We express the estimate of exercise intensity as a regression: given the breathing sound, mel spectrogram *m* and corresponding rope jumping speed *v*, *m* ∈ *M*, *v* ∈ *V*, we aim to learn a function *G*: *M* + *V* → *R*, where *M* and *R* denote the mel spectrogram and exercise intensity spaces, respectively. Since *m* is determined by *Q*(*z*, *r*, *s*), *z* is the breathing sound signal, *r* ∈ *R*, *s* ∈ *S*, and *S* contain domain characteristics of the current scene, such as user diversity and environmental diversity. For the estimation of exercise intensity, only the features induced by *z* and *r* are desired, while the features induced by *s* should be removed. Therefore, we used a deep neural network to approximate *G* and combine it with a domain adversarial adaptation to eliminate the effect of *s*.

Figure 8 illustrates the structure of our neural network, which consists of a feature extractor, an exercise intensity estimator, and a domain discriminator. The feature extractor transforms the input mel spectrogram and rope jumping speed into a feature vector [36], the exercise intensity estimator uses the feature vector as the input to evaluate the user’s exercise intensity, and the domain discriminator takes the weighted feature vector as input to identify different domains. The weight is obtained from the exercise intensity estimator. The network learns domain-independent features by jointly training a feature extractor and a domain discriminator. Specifically, the domain discriminator is trained to determine which domain each sample belongs to, while the feature extractor is trained to learn how to trick the domain discriminator into not making the correct determination so that the network can learn domain-independent features. It is because of the ability to obtain domain-independent features that the network has a strong migration capability, allowing it to adapt to different users and environments.

We trained the network using three sets of training data: (1) breathing sound mel spectrogram and its corresponding rope jumping speed set *D*, *D* = *M* + *V*, (2) corresponding ground truth exercise intensity set *R*, and (3) domain label set *T*. As exercise intensity is difficult to assess subjectively, we need a reliable indicator of the ground truth of exercise intensity. Heart rate is an ideal parameter for determining the intensity of exercise loads [37] and it is physiologically argued that heart rate is a realistic reflection of exercise intensity [38,39]. According to exercise physiology, exercise intensity is divided into three levels: small, medium, and large, corresponding to heart rates of 120–140 beats/min, 141–160 beats/min, and 161–180 beats/min. According to this criteria, we calculate the exercise intensity *r* as:

(21) $$r=\frac{h-h_{min}}{h_{max}-h_{min}}*100\%,$$

where *h* is the post-exercise heart rate, $h_{min}$ and $h_{min}$ are the minimum and maximum heart rates corresponding to the exercise intensity levels, respectively, as the exercise intensity ground truth.

The feature extractor consists of a ResNet18 network that maps the input to a lower-dimensional feature vector. The deep residual neural network (ResNet) can effectively avoid the problem of gradient explosion and gradient disappearance when a neural network reaches a certain number of layers, and can optimize the performance of the deep network, which has been widely used [40]. The exercise intensity estimator consists of a fully connected (FC) layer that infers the user’s exercise intensity based on the obtained feature vector. The domain discriminator also consists of an FC layer with a weighted feature vector as input [41]. This is because we expect the domain discriminator to recognize only different users and scenes; however, different exercise intensities produce distinctive features of the mel spectrogram. If we do not account for this feature, the domain discriminator may treat the exercise intensity as a domain. Therefore in order to exclude feature information on exercise intensity, the domain discriminator takes the weighted feature vector as input, where the weight is the exercise intensity derived by the exercise intensity estimator.

The training phase of the network entails two tasks: (1) achieving accurate prediction of the user’s exercise intensity and minimizing the estimation error of the exercise intensity estimator, (2) maximizing the classification error of the domain discriminator enables the network to extract domain-independent features. Therefore, the loss function of this network consists of two parts, the regression prediction loss of the exercise intensity estimator $L_{r}$ and the classification loss of the domain discriminator $L_{t}$. The total loss function of the network *L* can be expressed as:

(22) $$L\left(\theta_{f},\theta_{r},\theta_{t}\right)=L_{r}\left(\theta_{f},\theta_{r}\right)-\alpha L_{t}\left(\theta_{f},\theta_{t}\right),$$

where $\theta_{f}$, $\theta_{r}$, $\theta_{t}$ are the parameters of the feature extractor, exercise intensity estimator, and domain discriminator, respectively, and α is a positive hyperparameter used to control the effect of the domain classifier on the optimized feature extractor. The exercise intensity estimator is used to predict the user’s exercise intensity, which is a regression problem, so we use the mean square error as its loss function $L_{r}$:

(23) $$L_{r}\left(\theta_{f},\theta_{r}\right)=\frac{1}{R}\sum_{i=1}^{R}{\left(h_{i}-\hat{h_{i}}\right)}^{2},$$

where $h_{i}$ and $\hat{h_{i}}$ are the true and predicted exercise intensities of the *i*-th input sample, respectively. The domain discriminator is used to classify different domains, so its loss function $L_{t}$ uses the categorical cross-entropy loss function:

(24) $$L_{t}\left(\theta_{f},\theta_{t}\right)=-\frac{1}{D}\sum_{i=1}^{D}\sum_{j=1}^{T}\log\left(\hat{t_{i,j}}\right),$$

where $\hat{t_{i,j}}$ is the predicted probability that the *i*-th sample belongs to the *j*-th domain.

During training, the objectives for $\theta_{r}$ and $\theta_{t}$ are to minimize $L_{r}$ and $L_{t}$, respectively. According to (14), it can be seen that their relationship is adversarial; $\theta_{f}$ tricks the domain discriminator by maximizing $L_{t}$ so that *L* is minimized. This adversarial learning allows the feature extractor to extract domain-independent features that can characterize the exercise intensity well; thus, the network can easily adapt to different users and environments.

When a user completes a rope jumping workout, he/she can obtain his/her number of jump ropes, rope jumping speed, and exercise intensity. Based on the data, users will be able to further adjust their rope jumping schedule to achieve better workouts.

## 4. Implementation and Evaluation

In this section, we present the implementation details and evaluate the performance of our system.

### 4.1. Experiment Setup

We used two smartphones (i.e., Huawei Mate 40 Pro and iPhone 13 Pro Max) and two Bluetooth headphones to collect the acoustic signal. The experiments were conducted in three different environments: a quiet indoor area, a noisy indoor area with music, and a noisy outdoor area with traffic noise. The indoor areas include three rooms with different flooring materials (including wood, tile, and concrete floors). To evaluate the performance of the system, we recruit 8 volunteers (5 males and 3 females, aged from 20 to 40) for the experiment, each volunteer has 3 different choices in footwear (including rubber-soled sneakers, foam-soled sneakers, and slippers). Details of the volunteers’ data are given in Table 1. Eight volunteers are also typical for exercise monitoring studies [42]. In addition, we divided the subjects into three categories according to their self-report information, i.e., novice: volunteers who barely exercised (average exercise time per month was less than half an hour), normal: volunteers who exercised occasionally (average exercise time per month was more than half an hour and less than two hours), master: volunteers who exercised regularly (average exercise time per week was at least one hour). To obtain the ground truth of exercise intensity, each volunteer needed to wear a heart rate bracelet while rope jumping.

The researcher recorded the heart rate at the end of each rope jumping and calculated the exercise intensity according to (21).

In the attentive mechanism-based GAN, we used Adam with a learning rate of 1 × 10${}^{-4}$ for training until convergence. In order to perform the domain adversarial adaptation, we needed two choices for each of the three factors defining an experiment’s setting (i.e., user, floor material, and footwear), so we used eight settings for training.

For each chosen setting, we further split the collected samples into training and testing at a ratio of 3:1. The labels for the training and testing samples were made differently in order to fairly evaluate the prediction performance.

Three sets of experiments were deployed. We first evaluated the performance of the rope jumping count, considering the impacts of the rope jumping duration, environmental noise, rope jumping conditions (e.g., footwear and floor materials), rope jumping methods, and the diversity of users on the counting error, and compared the counting performance of our system with other existing jump rope counting systems. We then evaluated the performance of the exercise intensity, taking into account the impacts of both the environment and the user’s proficiency in rope jumping. Finally, we conducted several ablation experiments.

### 4.2. Performance of Rope Jumping Count

#### 4.2.1. Impact of Rope Jumping Duration and Environmental Noise

**Figure 9:**
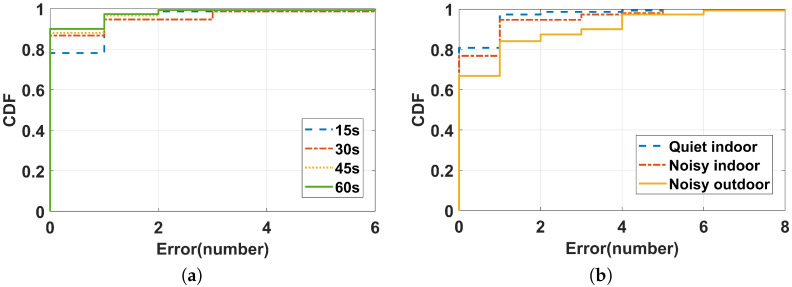
CDF of counting error with varied durations (**a**) (Performances for different durations) and environments (**b**) (Performance for different environments).

In the first set of experiments, we evaluated the performance of our proposed rope jumping intensity monitoring system with different rope jumping durations. Generally speaking, the length of a group of rope jumping in 60 s or less, which is the length of time to ensure the fitness effect and it will not cause damage to the body. Therefore, we evaluated the performance of rope jumping counts at different rope jumping durations (including 15 s, 30 s, 45 s, and 60 s) within 60 s. The cumulative distribution functions (CDFs) of the counting errors for different durations are plotted in Figure 9a. The mean count errors are 0.28, 0.27, 0.15, and 0.15, respectively. The error is greatest at a duration of 15 s, but 78% of the data have errors of 0. It can be seen that the error decreases with increasing rope jumping duration, this is because longer durations stabilize the rope jumping speed and the detected rope-jumping sound becomes more accurate. Combined with this result, we fixed a rope jumping duration of 30 s and tested the performances of the jump rope counting in different environments (including a quiet indoor area, a noisy indoor area with music, and a noisy outdoor area with traffic noise). Volunteers were required to perform 30 s rope-jumping exercises in different environments. As shown in Figure 9b, the average errors for the three environments were 0.25, 0.58, and 0.78, respectively. The results demonstrate the robustness of the rope jumping count to environmental noises. This demonstrates that our system can achieve a satisfactory performance even in noisy environments. Further, this figure also shows that better counting accuracy can be achieved in a quiet environment. This is natural because it is relatively easier to identify the rope-jumping sound from the noise in a relatively quiet environment.

#### 4.2.2. Impacts of Rope Jumping Conditions

We now explore the impacts of footwear and floor materials on the counting performance.

**Figure 10:**
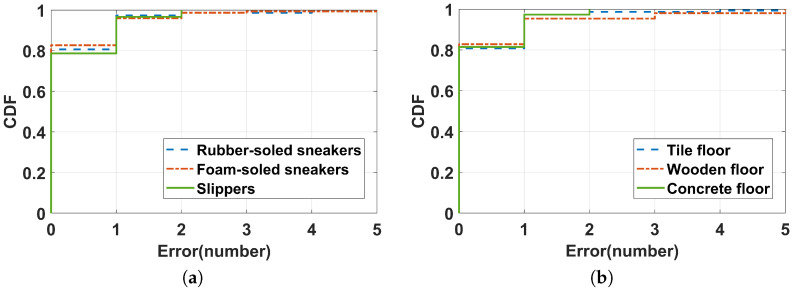
CDF of counting errors with various footwear (**a**) (Performances for different footwear) and floor material (**b**) (Performances on different floor materials).

We conducted the experiment according to the three footwear and floor materials explained in the experiment setup. Volunteers first wore three kinds of shoes to perform 30 s of rope-jumping exercises on the wood floor, then volunteers wore rubber-soled sports shoes to perform the same rope-jumping exercises on three different flooring materials. The results are shown in Figure 10b; footwear and floor materials virtually introduced no impact on counting. This is because the shoes and floor materials mainly affected the amplitude of the rope-jumping sound, while our system mainly uses the periodicity of the rope-jumping sound for counting and could dynamically set the threshold for rope-jumping sound detection according to the energy level of the acoustic signal; setting the dynamic threshold significantly improves the performance of the system.

#### 4.2.3. Impact of Rope Jumping Methods

**Figure 11:**
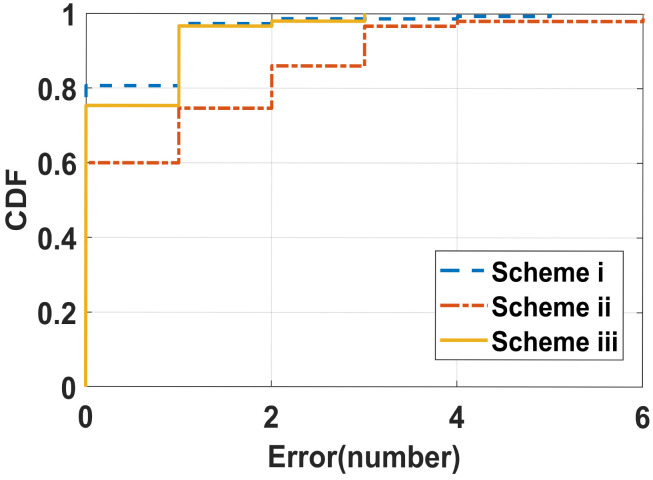
CDF of the counting error with various rope jumping methods.

Users may jump in different ways, e.g., alternate jumping on one foot and jumping on both feet. Therefore, we designed three rope jumping schemes (i) only jumping on both feet; (ii) alternate jumping on one foot; (iii) rope jumping with one foot alternating and then both feet. Volunteers performed a 30 s rope jumping exercise according to different schemes, where scheme iii required volunteers to rope jump with one foot for the first 10 s and then with both feet for the next 20 s. The CDFs of the average counting errors under scheme i, scheme ii, and scheme iii are plotted in Figure 11. The average errors of schemes i/ii/iii are 0.25, 0.86, and 0.54, respectively. It can be seen that our system has good performance. In addition, the average errors of schemes ii and iii are relatively higher than those of scheme i. The reason is that when rope jumping with one foot, the user’s body has an unstable center of gravity and the user needs to balance the body by adjusting the timing of the foot landing, resulting in a change in the jumping cycle.

#### 4.2.4. Impact of the Diversity of Users

**Figure 12:**
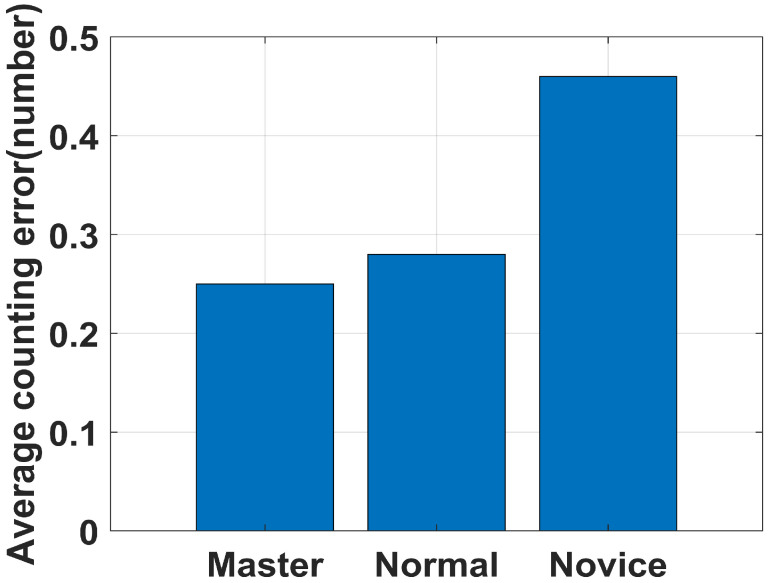
Average counting error with varied user.

Eight volunteers were involved in this experiment, with heights ranging from 1.6 to 1.88 m and weights ranging from 45 to 84 kg; it included people who rope jumped regularly, people who rope jumped occasionally, and people who rarely rope jumped. We divide them into three categories: master, normal, and novice. The average errors are shown in Figure 12. We can see that our system has good performance on both master and normal users. However, due to the fact that novices almost do not jump rope, they could not maintain a steady jumping rhythm when rope jumping led to a slightly higher counting error. On the whole, the system performs well under different rope jumping levels and body types.

#### 4.2.5. Average Counting Error Compared with Smart Rope, YaoYao, and TianTian

We compare the performances of existing jump rope counting systems. Volunteers performed a 30 s rope jumping exercise in the same environment using different systems/products (Smart Rope, YaoYao, TianTian, and our system), respectively. The results are shown in Table 2. Smart Rope and YaoYao performed well, with average errors of 1.8 and 2.6, respectively.

Both systems apply sensors for jump rope counting and some hand movements may affect the counting results, which resulted in an error of about 1–3. TianTian had the highest error, with an average error of 6.3. The reason is that TianTian uses a vision-based method to count jump ropes by recording the user’s hand-raising movements, and is, therefore, susceptible to interference from hand movements, the activities of surrounding people, and lighting conditions. Our system performs the best with an average error of 0.32, outperforming Smart Rope, TianTian, and YaoYao.

### 4.3. Performance of Spectrogram Optimization and Effect Evaluation

#### 4.3.1. Impact of Environmental Noise

**Figure 13:**
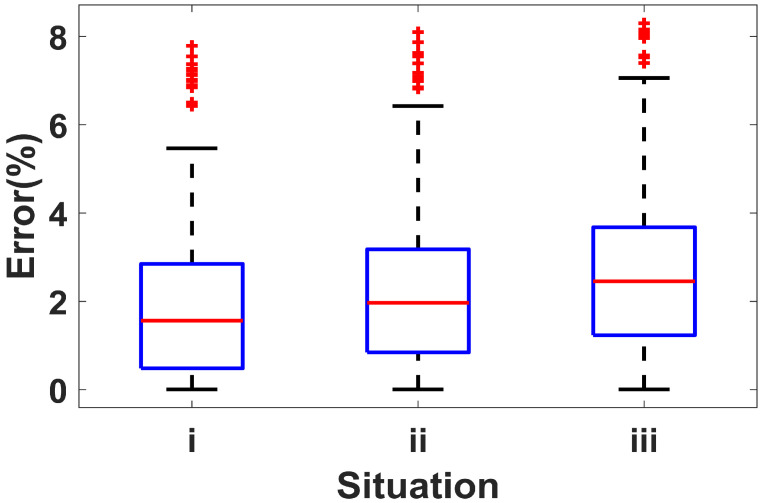
Box-plot evaluation error with varied situations. **i**: Only jumping on both feet; **ii**: Alternate jumping on one foot; **iii**: Rope jumping with one foot alternating and then both feet.

We evaluated the performance of our proposed exercise intensity monitoring system in different environments (i.e., the quiet indoor area, the noisy indoor area with music, and the noisy outdoor area with traffic noise). The box-plot evaluation error is shown in Figure 13. The evaluation error increases in noisy environments; in all cases, the average error is less than 2.83%. The results demonstrate the robustness of our system to noise. This advantage of our system is contributed to by, first, the band-pass filter eliminates high-frequency noises, second, the attentive mechanism-based GAN can generate optimized spectrograms that mitigate noise interference, and third, the domain adaptation can extract environment-independent features.

#### 4.3.2. Impact of the Diversity of Users

**Figure 14:**
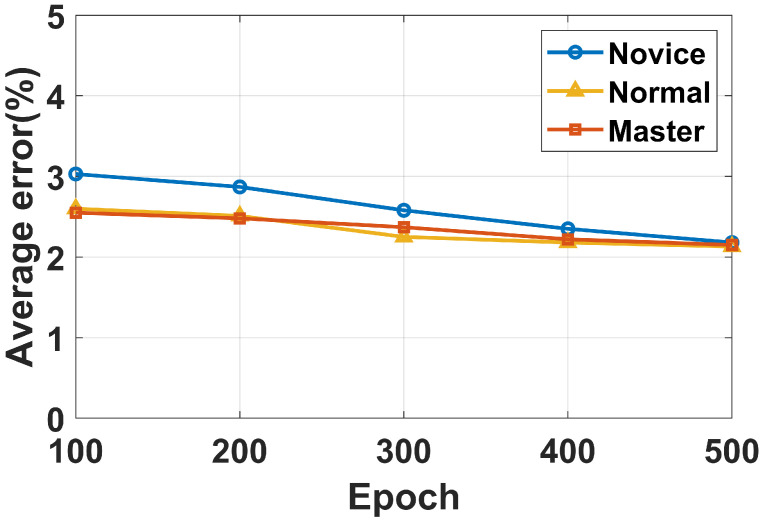
Average evaluation error with varied user.

Figure 14 shows the average error for eight volunteers at different levels. We can see that the system has good performance with both normal and master users, this is because volunteers with exercise bases have more stable breathing after exercise. However, due to the fact that novices hardly ever exercise, they breathe more sharply after exercise, leading to higher errors. However, after a few training sessions, the evaluation errors of novice users can reach normal levels. Overall, the system performs well for different levels of exercise. The reasons are that domain adaptation can extract user-independent features and the system uses the rope jumping speed and breathing sound for a joint evaluation, applying data from multiple modalities, which allows for more robust predictions to be made.

### 4.4. Ablation Study

To demonstrate the effectiveness of each component proposed in Section 3, we conducted several ablation experiments [43]. Specifically, we designed three experiments by removing the components of the attention mechanism, local discriminator, and domain adaptation, respectively. The experimental results are shown in Table 3.

It can be observed that, with the addition of the attention mechanism and local discriminator, the performance of the algorithm is significantly improved, and the average prediction error is reduced by 1%. In addition, the average error can reach 2.3% by adding domain adaptation; without domain adaptation, the average prediction error would reach 5.8%. These results strongly confirm that our system network offers a robust cross-domain exercise intensity prediction performance.

## 5. Conclusions and Future Work

In this paper, we propose a system for rope jumping exercise monitoring using a smartphone and headphones. Our system utilizes headphones to capture the rope-jumping sound to count the number of rope jumps, addressing the significant challenge of separating the rope-jumping sounds, which is critical for jump rope counting. Furthermore, our system extracts features from the rope jumping speed and the mel spectrograms of the user’s breathing sound to predict the exercise intensity. The network utilizes attentive mechanism-based GAN to optimize the breathing sound mel spectrogram to improve prediction accuracy and employs domain adaptation to migrate to different users and environments. The extensive experiments show that our proposed rope jump monitoring system is robust under different conditions. Therefore, we deem it feasible to use smartphones and headphones for rope jumping exercise monitoring.

Our system is suitable for single-person rope jumping. When multiple users perform rope-jumping exercises at the same time, there may be a large counting error due to the mutual influence of the rope-jumping sounds between users. As part of our future work, we will explore more unique characteristics of the rope-jumping sound to identify different rope-jumping rhythms of different users and use deep learning to identify multiple users automatically.

## Figures and Tables

**Table 1:** Demographics of Volunteer.

ID	Gender	Age	Proficiency
1	Female	20–25	Master
2	Female	20–25	Normal
3	Male	20–25	Master
4	Male	25–30	Novice
5	Female	25–30	Normal
6	Male	25–30	Novice
7	Female	30–35	Novice
8	Female	30–35	Novice

**Table 2:** Results of the average counting errors for different systems.

	Smart Rope	YaoYao	TianTian	Our System
Average counting error in 30 s (number)	1.8	2.6	6.3	0.32

**Table 3:** Results of ablation experiments.

Baseline Network	Attention Mechanism	Local Discriminator	Domain Adaptation	Average Prediction Error
*√*				5.8%
*√*		*√*	*√*	3.5%
*√*	*√*		*√*	3.9%
*√*	*√*	*√*		4.8%
*√*	*√*	*√*	*√*	2.3%

## Data Availability

Not applicable.

## Abbreviations

The following abbreviations are used in this manuscript:

GAN	generative adversarial network
LSGAN	least-square generative adversarial network
ResNet	residual neural network
FC	fully connected

## References

[1] Seals D.R., Nagy E.E., Moreau K.L. (2019). Aerobic exercise training and vascular function with ageing in healthy men and women. J. Physiol..

[2] Mikkelsen K., Stojanovska L., Polenakovic M., Bosevski M., Apostolopoulos V. (2017). Exercise and mental health. Maturitas.

[3] Campbell B.I., Bove D., Ward P., Vargas A., Dolan J. (2017). Quantification of Training Load and Training Response for Improving Athletic Performance. Strength Cond. J..

[4] Halson S.L. (2014). Monitoring Training Load to Understand Fatigue in Athletes. Sport. Med..

[5] Giordano P., Mautone A., Montagna O., Altomare M., Demattia D. (2017). Training Load and Fatigue Marker Associations with Injury and Illness: A Systematic Review of Longitudinal Studies. Sport. Med..

[6] Jin X., Yao Y., Jiang Q., Huang X., Zhang J., Zhang X., Zhang K. Virtual Personal Trainer via the Kinect Sensor. Proceedings of the IEEE International Conference on Communication Technology.

[7] Khanal S.R., Fonseca A., Marques A., Barroso J., Filipe V. Physical exercise intensity monitoring through eye-blink and mouth’s shape analysis. Proceedings of the 2018 2nd International Conference on Technology and Innovation in Sports, Health and Wellbeing (TISHW).

[8] Wu B.F., Lin C.H., Huang P.W., Lin T.M., Chung M.L. A contactless sport training monitor based on facial expression and remote-PPG. Proceedings of the 2017 IEEE International Conference on Systems, Man, and Cybernetics (SMC).

[9] Seeger C., Buchmann A., Laerhoven K.V. myHealthAssistant: A Phone-based Body Sensor Network that Captures the Wearer’s Exercises throughout the Day. Proceedings of the 6th International ICST Conference on Body Area Networks.

[10] Tang W., He F., Liu Y. (2022). YDTR: Infrared and visible image fusion via y-shape dynamic transformer. IEEE Trans. Multimed..

[11] Ding H., Shangguan L., Yang Z., Han J., Zhou Z., Yang P., Xi W., Zhao J. FEMO: A platform for free-weight exercise monitoring with RFIDs. Proceedings of the 13th ACM Conference on Embedded Networked Sensor Systems.

[12] Morris D., Saponas T.S., Guillory A., Kelner I. RecoFit: Using a wearable sensor to find, recognize, and count repetitive exercises. Proceedings of the SIGCHI Conference on Human Factors in Computing Systems.

[13] Shen C., Ho B.J., Srivastava M. (2018). MiLift: Efficient Smartwatch-Based Workout Tracking Using Automatic Segmentation. IEEE Trans. Mob. Comput..

[14] Xie Y., Li F., Wu Y., Wang Y. HearFit: Fitness Monitoring on Smart Speakers via Active Acoustic Sensing. Proceedings of the IEEE INFOCOM 2021-IEEE Conference on Computer Communications.

[15] Zhu Y., Wang D., Zhao R., Zhang Q., Huang A. FitAssist: Virtual fitness assistant based on WiFi. Proceedings of the 16th EAI International Conference on Mobile and Ubiquitous Systems: Computing, Networking and Services.

[16] Chiang T.H., Chuang Y.T., Ke C.L., Chen L.J., Tseng Y.C. Calorie Map: An Activity Intensity Monitoring System Based on Wireless Signals. Proceedings of the 2017 IEEE Wireless Communications and Networking Conference (WCNC).

[17] Wang W., Kumar N., Chen J., Gong Z., Kong X., Wei W., Gao H. (2020). Realizing the Potential of the Internet of Things for Smart Tourism with 5G and AI. IEEE Netw..

[18] Pernek I., Hummel K.A., Kokol P. (2012). Exercise repetition detection for resistance training based on smartphones. Pers. Ubiquitous Comput..

[19] Ledig C., Theis L., Huszár F., Caballero J., Cunningham A., Acosta A., Aitken A.P., Tejani A., Totz J., Wang Z. Photo-Realistic Single Image Super-Resolution Using a Generative Adversarial Network. Proceedings of the 2017 IEEE Conference on Computer Vision and Pattern Recognition (CVPR).

[20] Yang S., Wang Z., Wang Z., Xu N., Guo Z. Controllable artistic text style transfer via shape-matching gan. Proceedings of the IEEE/CVF International Conference on Computer Vision.

[21] Zhu J.-Y., Park T., Isola P., Efros A.A. Unpaired image-to-image translation using cycle-consistent adversarial networks. Proceedings of the IEEE International Conference on Computer Vision.

[22] Tangram Factory Inc (2015). Smart Rope. http://tangramfactory.com/smartrope/en.

[23] Wang J. (2017). YaoYao. https://yy.onlytalk.top.

[24] Epstein L.H., Paluch R.A., Kalakanis L.E., Goldfield G.S., Cerny F.J., Roemmich J.N. (2001). How Much Activity Do Youth Get? A Quantitative Review of Heart-Rate Measured Activity. Pediatrics.

[25] Shanghai Littlelights Education Technology Co., Ltd (2020). TianTian. https://www.tiantiantiaosheng.com.

[26] Wang W., Chen J., Wang J., Chen J., Liu J., Gong Z. (2020). Trust-Enhanced Collaborative Filtering for Personalized Point of Interests Recommendation. IEEE Trans. Ind. Informatics.

[27] Giannakopoulos T. (2009). A Method for Silence Removal and Segmentation of Speech Signals, Implemented in Matlab. Ph.D. Thesis.

[28] Mahdy A.M.S., Lotfy K., El-Bary A.A. (2022). Use of optimal control in studying the dynamical behaviors of fractional financial awareness models. Soft Comput..

[29] Mahdy A.M.S. (2022). A numerical method for solving the nonlinear equations of Emden-Fowler models. J. Ocean. Eng. Sci..

[30] Guo Q., Ji G., Li N. A improved dual-threshold speech endpoint detection algorithm. Proceedings of the 2010 2nd International Conference on Computer and Automation Engineering (ICCAE).

[31] Nicolò A., Massaroni C., Schena E., Sacchetti M. (2020). The Importance of Respiratory Rate Monitoring: From Healthcare to Sport and Exercise. Sensors.

[32] Nicolò A., Girardi M., Bazzucchi I., Felici F., Sacchetti M. (2018). Respiratory frequency and tidal volume during exercise: Differential control and unbalanced interdependence. Physiol. Rep..

[33] Ronneberger O., Fischer P., Brox T. (2015). U-Net: Convolutional networks for biomedical image segmentation. Medical Image Computing and Computer-Assisted Intervention 2015.

[34] Mao X., Li Q., Xie H., Lau R.Y., Wang Z., Paul Smolley S. Least squares generative adversarial networks. Proceedings of the IEEE International Conference on Computer Vision.

[35] Ulyanov D., Vedaldi A., Lempitsky V. Improved texture networks: Maximizing quality and diversity in feed-forward stylization and texture synthesis. Proceedings of the IEEE Conference on Computer Vision and Pattern Recognition.

[36] Wang W., Yu X., Fang B., Zhao D.-Y., Chen Y., Wei W., Chen J. (2022). Cross-modality LGE-CMR Segmentation using Image-to-Image Translation based Data Augmentation. IEEE/ACM Trans. Comput. Biol. Bioinform..

[37] Strath S.J., Swartz A.M., Bassett D.R., O’Brien W.L., King G.A., Ainsworth B.E. (2000). Evaluation of heart rate as a method for assessing moderate intensity physical activity. Med. Sci. Sport. Exerc..

[38] Myles W.S., Dick M.R., Jantti R. (1981). Heart rate and rope skipping intensity. Res. Q. Exerc. Sport.

[39] Chen J., Sun S., Zhang L.-B., Yang B., Wang W. (2021). Compressed Sensing Framework for Heart Sound Acquisition in Internet of Medical Things. IEEE Trans. Ind. Inform..

[40] He K., Zhang X., Ren S., Sun J. Deep residual learning for image recognition. Proceedings of the IEEE Computer Society Conference on Computer Vision and Pattern Recognition (CVPR).

[41] Cai C., Pu H., Wang P., Chen Z., Luo J. (2021). We Hear Your PACE: Passive Acoustic Localization of Multiple Walking Persons. Proc. ACM Interact. Mob. Wearable Ubiquitous Technol..

[42] Ren Y., Zheng Z., Liu H., Chen Y., Wang C. Breathing Sound-based Exercise Intensity Monitoring via Smartphones. Proceedings of the 2021 International Conference on Computer Communications and Networks (ICCCN).

[43] Jiang Y., Gong X., Liu D., Cheng Y., Fang C., Shen X., Yang J., Zhou P., Wang Z. (2021). EnlightenGAN: Deep Light Enhancement Without Paired Supervision. IEEE Trans. Image Process..

